# Mithramycin A Inhibits Colorectal Cancer Growth by Targeting Cancer Stem Cells

**DOI:** 10.1038/s41598-019-50917-3

**Published:** 2019-10-23

**Authors:** Waise Quarni, Rinku Dutta, Ryan Green, Sandhyabanu Katiri, Bhaumik Patel, Shyam S. Mohapatra, Subhra Mohapatra

**Affiliations:** 1 Department of Molecular Medicine, Morsani College of Medicine, University of South Florida, Tampa, FL 33612 USA; 2 Department of Internal Medicine, Morsani College of Medicine, University of South Florida, Tampa, FL 33612 USA; 3 Center for Research and Education in Nanobioengineering, Morsani College of Medicine, University of South Florida, Tampa, FL 33612 USA; 4 James A Haley VA Hospital, Tampa, FL 33612 USA; 5 Hunter Holmes McGuire VA Medical Center, Richmond, VA 23249 USA; 6 Division of Hematology, Oncology, and Palliative Care, Department of Internal Medicine and Massey Cancer Center, Virginia Commonwealth University, Richmond, VA 23298 USA

**Keywords:** Cancer stem cells, Colon cancer

## Abstract

The pivotal role of cancer initiating stem cells (CSCs) in tumor initiation, growth, metastasis and drug resistance has led to the postulation of a ‘total cancer therapy’ paradigm, which involves targeting both cancer cells and CSCs for effective therapy. However, the progress in identifying drugs for total cancer therapy has been limited. Herein, we show for the first time that mithramycin A (Mit-A) can successfully inhibit CSC proliferation, in addition to inhibiting bulk cancer cells in a model of colorectal cancer (CRC), the second leading cause of death among men and women in the United States. To this end, a polymeric nanofiber scaffold culture system was established to develop 3D tumor organoids (tumoroids) from CRC cell lines such as HT29, HCT116, KM12, CT26 and MC38 as well as *ex vivo* mouse tumors. These tumoroids possessed increased expression of CSC markers and transcription factors, expanded the number of CSCs in culture and increased CSC functional properties measured by aldehyde dehydrogenase activity. Screening of an NCI library of FDA approved drugs led to the identification of Mit-A as a potential total cancer therapy drug. In both sphere and tumoroid culture, Mit-A inhibits cancer growth by reducing the expression of cancer stemness markers. In addition, Mit-A inhibits the expression of SP1, a previously known target in CRCs. Moreover, Mit-A significantly reduces growth of tumoroids in *ex vivo* cultures and CRC tumor growth *in vivo*. Finally, a dose-dependent treatment on CRC cells indicate that Mit-A significantly induces the cell death and PARP-cleavage of both CSC and non-CSC cells. Taken together the results of these *in vitro*, *ex vivo* and *in vivo* studies lead to the inference that Mit-A is a promising drug candidate for total cancer therapy of CRCs.

## Introduction

Colorectal cancer (CRC) is the third most common cancer in men, the second most common in women and the second most common cause of cancer-related deaths in the USA with an estimated 5% lifetime risk^1,2^. Particularly, patients with stage IV CRC have a 5-year survival rate of 10–14%^3^. Patients with advanced metastasized CRC tumors are often limited to traditional chemotherapies as their only treatment option. Current conventional chemotherapy of CRC includes 5-Fluorouracil (FU), FOLFOX and platinum-based treatment which shows a less than satisfactory efficacy in cancer patients^4^. The re-emergence of CRC after initial chemotherapy poses a major challenge in more than 50% of cases^5^. Therefore, finding a better and more effective chemotherapeutic drug to treat advanced metastatic CRCs remains a major unmet need.

A major drawback of currently practiced chemo, radio- and immune-therapies for cancers is that these therapies aim to reduce tumor burden by effectively killing tumor cells, but do not kill cancer-initiating stem cells (CSC)^6^. CSCs represent a small subset of tumor cells, which play pivotal roles in tumor initiation, growth, development of drug resistance and metastasis and contribute to the self-renewal and differentiation of diverse cell types in the tumor that form the tumor bulk^7,8^. The CSC phenotype in human colon cancer is associated with poor prognosis^1,9,10^. CSCs are responsible for the highly aggressive and invasive form of the disease that develops following chemo- and/or radiotherapy and hence, eliminating CSCs is likely to improve the survival rate in patients.

Current therapeutic approaches used to target CSCs focus on either inhibition of self-renewal or induction of differentiation^7,11^. A small percentage of CSCs in tumors has been a major barrier in CSC-targeted drug discovery^7,8^. To address this limitation, we developed a 3D nanofiber scaffold platform that allows tumor cells to develop tumor organoids (tumoroids), which mimics *in vivo* tumorigenesis^12–14^.These tumoroids significantly expand CSCs, which in turn has provided a new avenue for anti-CSC drug discovery^14^.

We reasoned that certain cancer drugs, in addition to their anti-cancer cell activity, might also possess anti-CSC activity and thus these drugs might provide ‘total cancer treatment’, i.e., these may kill both cancer cells and CSCs. We screened a library of FDA-approved drugs using the tumoroid culture method and identified mithramycin-A (Mit-A) as a potential CSC inhibitor. Mit-A is a potent anti-cancer drug which is being used to treat myeloid leukemia and testicular carcinoma^15,16^. A recent study suggests that it is also a potential chemotherapeutic drug to be used against cervical cancer^17^. Mit-A is a polyketide antibiotic which binds to the minor groove of DNA and inhibits transcription factor-DNA binding^18,19^. It is also known as a potent inhibitor of specificity protein 1 (SP1), which is involved in chemoresistant cancers^20^. However, the details of its mechanism of action in CRC cell killing and its potential role in targeting CSCs remain unclear.

In the current study, we have established a tumoroid culture system for CRC cells and examined the expansion of CSCs in this culture. Further, we investigated whether Mit-A can inhibit cell viability across different human and mouse colon cancer tumoroids cultured *in vitro* and *ex vivo* and in mouse models. The results of these studies demonstrated for the first time that Mit-A specifically targets CSCs and Mit-A is more effective in inhibiting CSC proliferation than other currently known chemo drugs used for treating CRCs.

## Results

### Tumoroid culture of colorectal cancer cell lines expands CSCs

**Figure 1:**
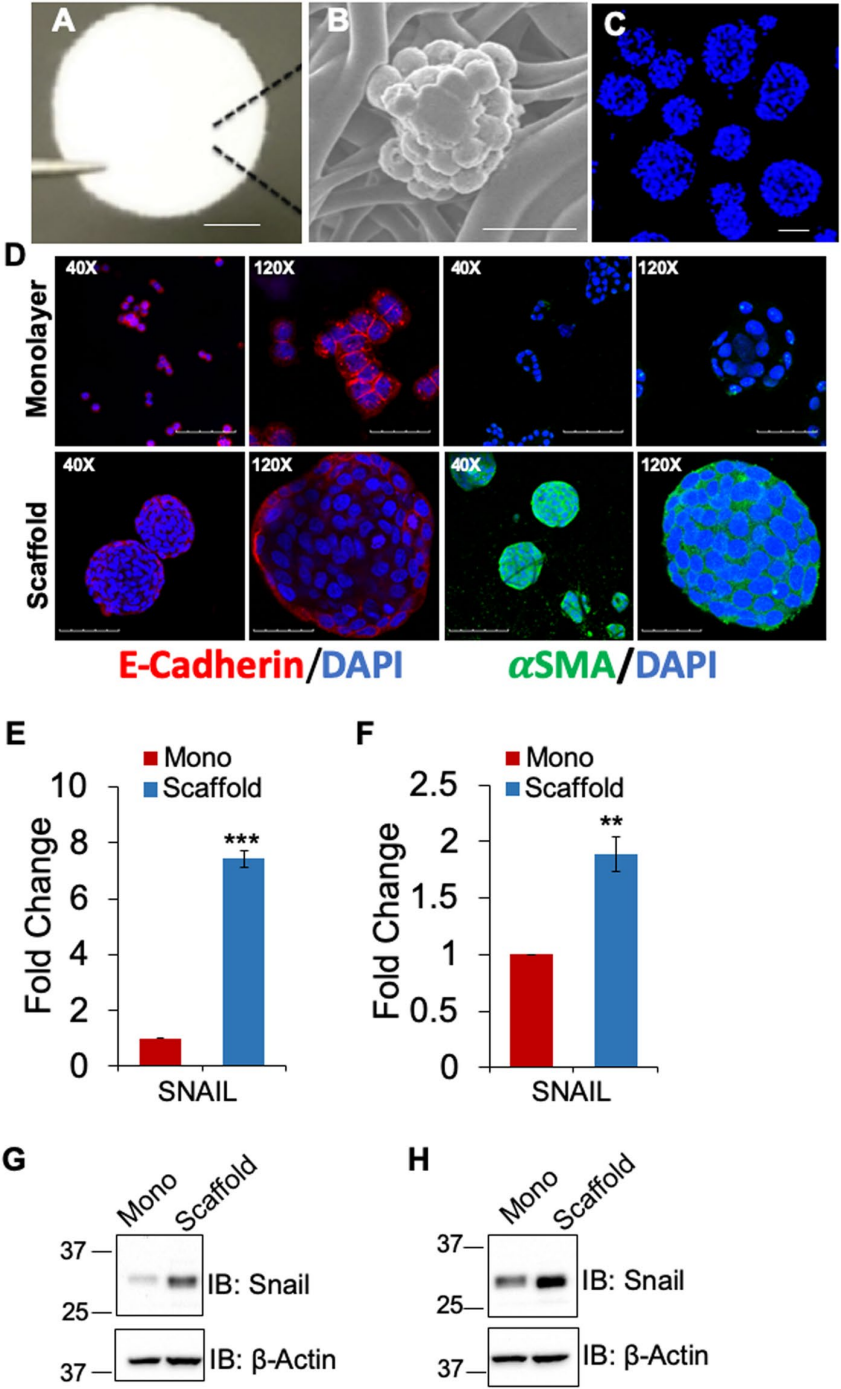
HT-29 tumoroids with features of EMT. (**A**) Scaffold matrix held by forceps tip, scale bar 1.6 mm. (**B**) Scanning EM of Day 4 HT-29 tumoroid on the scaffold, scale bar 20 µm. (**C**) Fluorescence micrographs of HT29 cells cultured on 3D scaffold. HT29 cells grown on scaffolds for 6 days and stained with Nuc-blue reagent, scale bar 100 µm. (**D**) IF staining of E-cadherin (red) and α-SMA (green) in HT-29 monolayer vs. tumoroids. Nuclei are DAPI (blue) stained, scale bars represent 100 µm (40X) and 30 µm (120X). Expression of SNAIL was assessed via qRT-PCR (**E**,**F**) and Western blot (**G**,**H**) in HT-29 and HCT-116 cells, respectively. *P < 0.05, **P < 0.01, and ***P < 0.001.

Previously, we reported that breast cancer cells cultured on 3D polymeric nanofiber scaffold (Fig. 1A) form tumoroids, which substantially (at least 5-fold) expand CSCs as determined by CSC biomarker expression and activity of aldehyde dehydrogenase enzyme (ALDH)^14^. Since CSC expansion of CRC tumoroids is hitherto unknown, we cultured three human CRC cells lines, HT29 (p53 mutant, K-RAS wild type, microsatellite stable), HCT116 (p53 wild-type, K-RAS mutant, microsatellite instable) and KM12 (p53 mutant, K-RAS wild type, microsatellite instable)^21^, and CT-26 murine cancer cells (p53 wild-type, K-RAS mutant, microsatellite stable)^22^ on 3D scaffold for 6 days and examined tumoroids for stemness markers by qPCR and flow cytometry. HT29 cells formed tumoroids when grown on the scaffold for 6 days (Fig. 1B,C). The SEM image showed typical tumoroid formation with a smooth surface and tight cell junctions (Fig. 1B). Nuc-blue stained HT-29 tumoroids are shown in Fig. 1C. To determine whether tumoroids formed on scaffold could undergo the epithelial to mesenchymal transition (EMT), we compared the HT-29 cells grown on monolayer vs. scaffold for expression of E-cadherin (epithelial marker) and αSMA (α smooth muscle actin) (mesenchymal marker). Immunofluorescence (IF) staining showed that over six days of culture, HT-29 tumoroids showed robust expression of αSMA but not E-cadherin. On the contrary, monolayer culture expressed E-cadherin but not αSMA (Fig. 1D). In addition, expression of the mesenchymal EMT marker, Snail, was also increased at both RNA and protein level in scaffold culture of HT-29 and HCT-116 compared to cells grown on monolayer (Fig. 1E–H). These results suggest that HT-29 tumoroids induced EMT when cultured on the scaffold.

**Figure 2:**
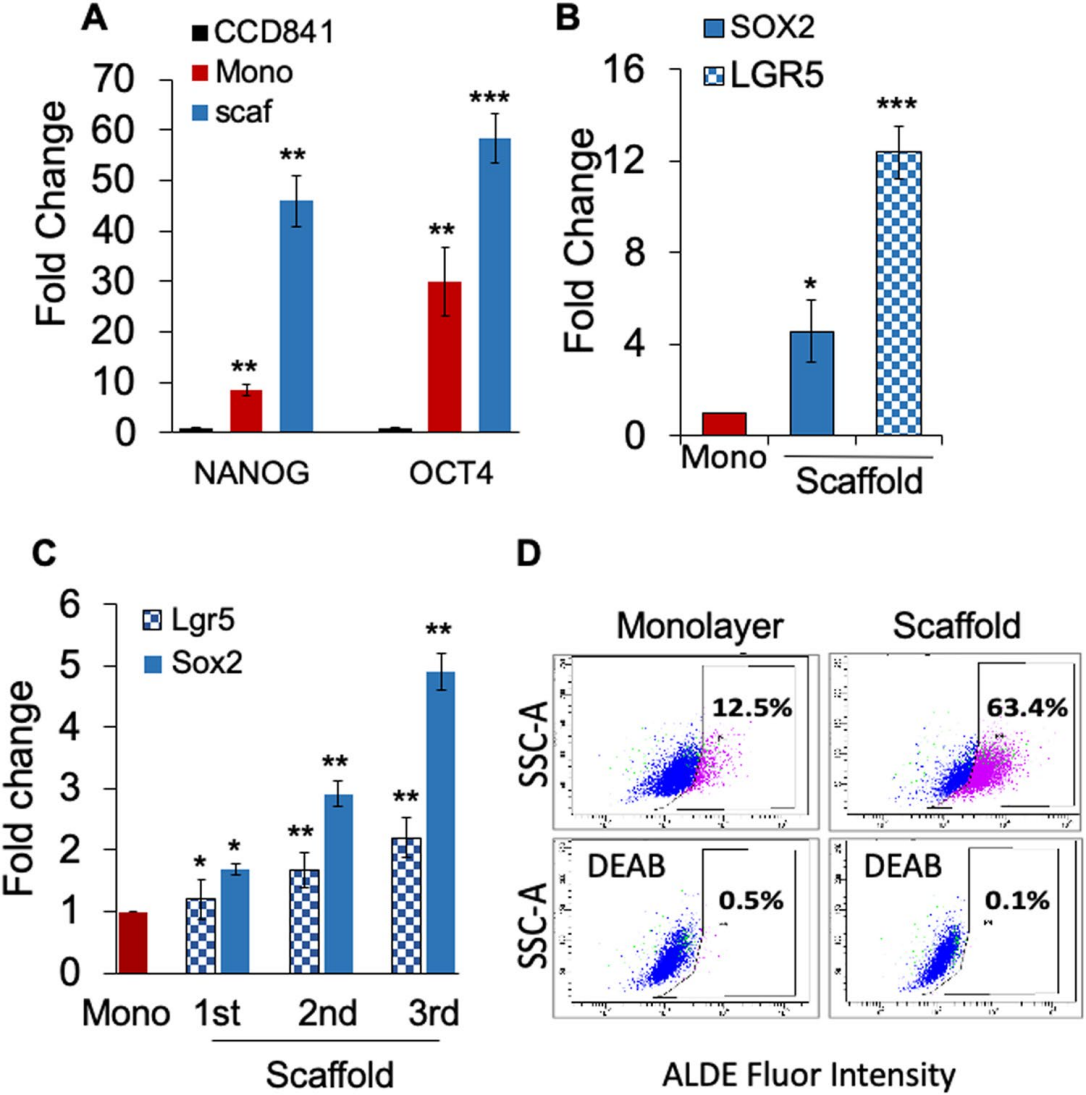
Tumoroid Culture Expands Cancer Stem Cells. (**A**–**C**) Cells were cultured on scaffold. On day 6, RNA was isolated from cell pellets, and CSC marker gene expression in monolayer vs scaffold cultures was assayed by qPCR. (**A-B**) Comparison of normal human colon cells CCD841 to CRC cells, HT29 grown on monolayer and scaffold, (**C**) CT26 grown on monolayer and scaffolds for 3 consecutive passages. (**D**) ALDH activity of HCT116 monolayer and tumoroids was measured using the ALDE Fluor kit (Stem Cell Technologies). Baseline fluorescence was calculated using DEAB. *p < 0.05; **p < 0.01; ***p < 0.001. A representative of three experiments shown.

Moreover, HT-29 tumoroid culture showed 50- and 60-fold higher expression of transcription factors NANOG and OCT4, respectively, compared to cells derived from normal colon, CCD841, and 5- and 2-fold compared to monolayer cells (Fig. 2A). Also, HT-29 tumoroids showed a 12- and 5-fold increase in transcription of LGR5 and stem cell related transcription factor SOX2, respectively, compared to monolayer (Fig. 2B). Further, tumoroids derived from CT-26 cells exhibited a gradual increase in expression of Lgr5 and Sox2 transcripts, as the tumoroids were successively passaged from P1 to P3 (Fig. 2C) in the 3D scaffold environment. In addition, tumoroids derived from HCT116 human colon cancer cells showed a 5-fold increase in ALDH activity (63.4%), which is a hallmark of stemness measurable by the aldefluor assay, over monolayer cells (12.5%) (Fig. 2D). These studies suggest that the scaffold induces increased expression of CSC markers and transcription factors both in human and mouse CRC cells.

### Identification of Mit-A as an inhibitor of CRC CSC and tumoroid growth

**Figure 3:**
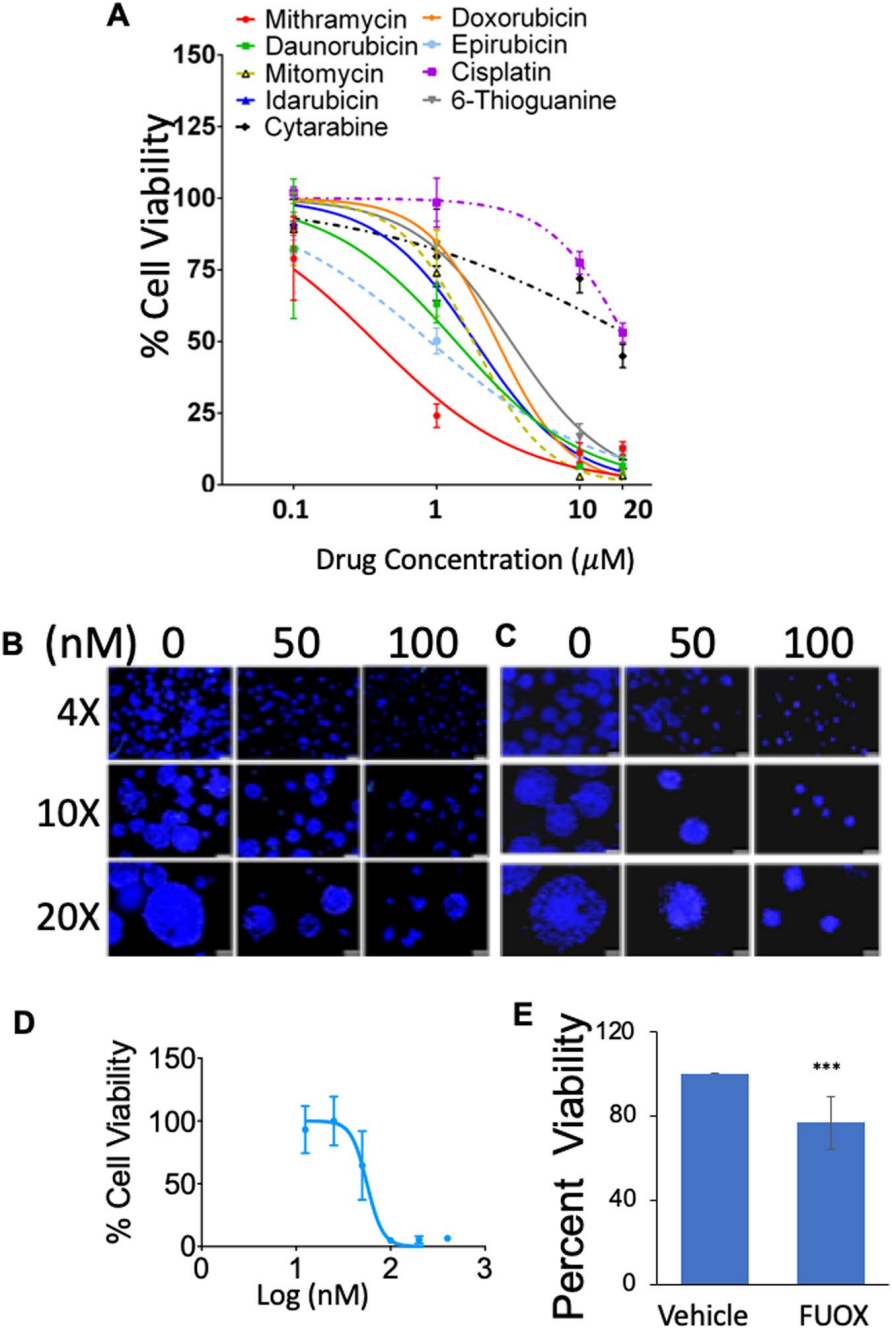
Mit-A is a potential anti-cancer drug. (**A**) HT-29 cells (5000/well) were cultured on scaffold for 4 days and then tumoroids were treated with varying concentrations of indicated drugs for 48 hours. Cell viability was assayed on day 6 by CellTiter-Glo (Promega). Percent viability was plotted using GraphPad Prism. A representative of 3 experiments shown. (**B**,**C**) HT29 and HCT116 cells, respectively, were grown on scaffolds for 4 consecutive days and then treated with Mit-A with indicated concentrations for additional 48 hours. Cells were stained with Nuc-blue and pictures were taken with a fluorescent microscope of different magnifications. Scale bar represents 200 µm, 100 µm and 50 µm for 4X, 10X and 20X magnifications, respectively. (**D**) KM12 cells were grown on scaffolds and treated with Mit-A for 48 hours. Cell viability was assayed on day 6 by CellTiter-Glo (Promega). (**E**) Day 4 HT-29 tumoroids were treated with vehicle or FUOX and cell viability was assessed on day 6 by CellTiter-Glo (Promega).

In the search for drugs that enable ‘total cancer therapy’, we screened an NCI Oncology Drugs Set of FDA-approved drugs using tumoroid platform in HT29 cells. Then, we selected the top nine drugs as candidate CSC inhibitors and assayed their ability to decrease viability of CRC CSCs using HT29 tumoroids. The tumoroids were treated for 48 hours with different drug candidates including cisplatin. Of these, Mit-A was found to have the lowest IC50 of all drugs tested (in the nanomolar range) (Fig. 3A). Thus, Mit-A was considered to be the most effective against HT29 cells.

In order to understand the mechanism of Mit-A inhibition of tumoroid growth, we cultured and treated tumoroids derived from HT-29, HCT-116 and KM12 cells. As shown in Fig. 3B–D, 48 hours of Mit-A treatment inhibited the growth of tumoroids. Both the number and size of HT-29, HCT116 and KM12 tumoroids were decreased in a dose-dependent manner. However, only 20% growth inhibition was observed when HT29 tumoroids were treated with a mixture of 25 µM of 5-FU and 0.625 µM of Oxaliplatin (FUOX) (Fig. 3E).

**Figure 4:**
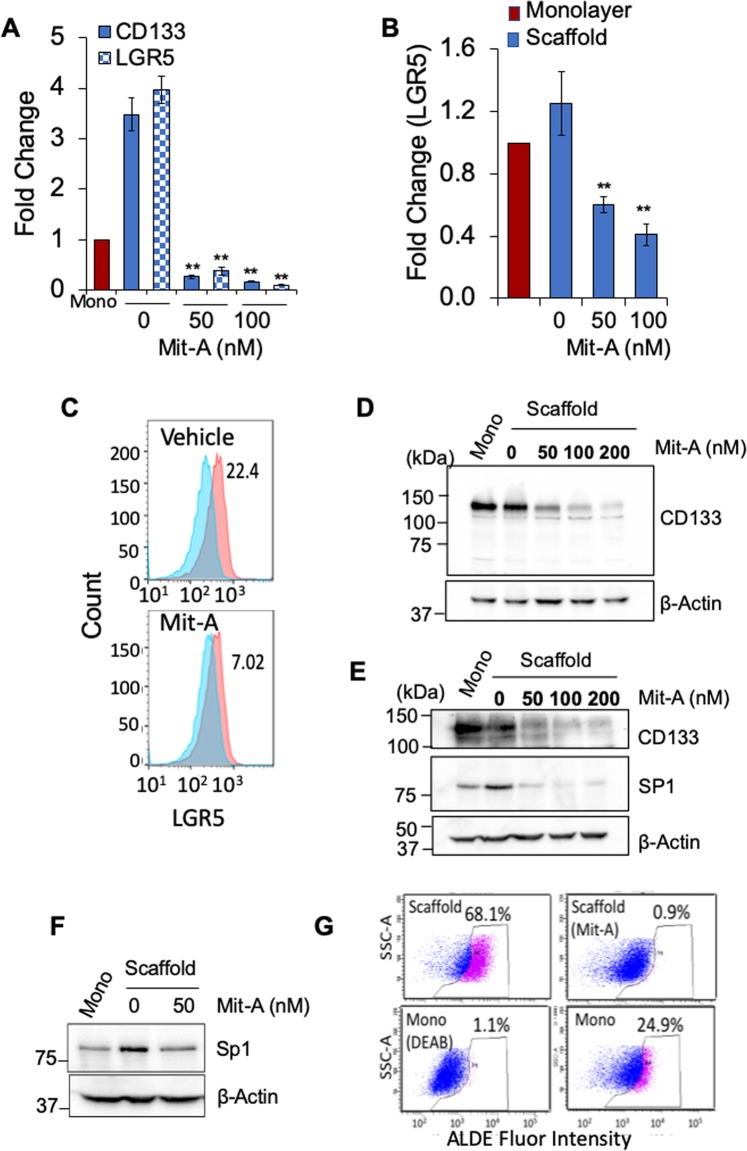
Mit-A targets CSCs. (**A**–**G**) Cells were cultured on scaffold as described earlier and examined on day 6. RNA was isolated from cell pellets, and CD133 and LGR5 gene expression in monolayer vs. 3D scaffold cultures of HT29 (**A**) and HCT116 (**B**) was assayed by qPCR. Cells were collected on day 6, LGR-5 expression in HCT-116 tumoroids exposed to Mit-A (100 nM) for 48 hrs quantified by flow cytometry (**C**). Expression of CD133 and Sp1 in HT29 (**D**,**F**) and HCT116 (**E**) was assessed by Western blotting. ALDH activity in HT-29 cells cultured on scaffold in the presence of Mit-A (100 nM) (**G**). Baseline fluorescence was calculated using DEAB. **p < 0.01. A representative of two experiments is shown.

To compare whether these physiological features were due to changes in stemness, we analyzed the expression of stemness markers through qPCR, flow cytometry and/or Western blotting. Both HT-29 and HCT-116 tumoroids showed a decrease in stemness factors when treated with Mit-A. Interestingly, we found that Mit-A inhibited CD133 expression in a dose-dependent manner in both cells by qPCR and Western blotting (Fig. 4A,D,E). Both qPCR and flow cytometry analyses showed that Mit-A significantly decreased expression of LGR5 in HT-29 and HCT116 (Fig. 4A,B,C). It was already known that Mit-A can inhibit cell growth through inhibition of SP1, a stemness-related transcription factor^17^. We tested whether Mit-A can also inhibit SP1 in CRC cells. Mit-A treatment was found to inhibit the SP1 expression in HCT116 and HT29 cells, respectively (Fig. 4E,F). Lastly, flow cytometry analysis showed that HT29 tumoroids showed a 2.5-fold increase in ALDH activity over monolayer, and Mit-A treatment significantly inhibited ALDH activity (decreased from 68.1% to 0.9% in tumoroids) (Fig. 4G). These data clearly indicate that Mit-A can inhibit tumoroid growth in part by suppressing the stemness marker expression in CRC cells.

### Mit-A can suppress cancer stemness in spheres

**Figure 5:**
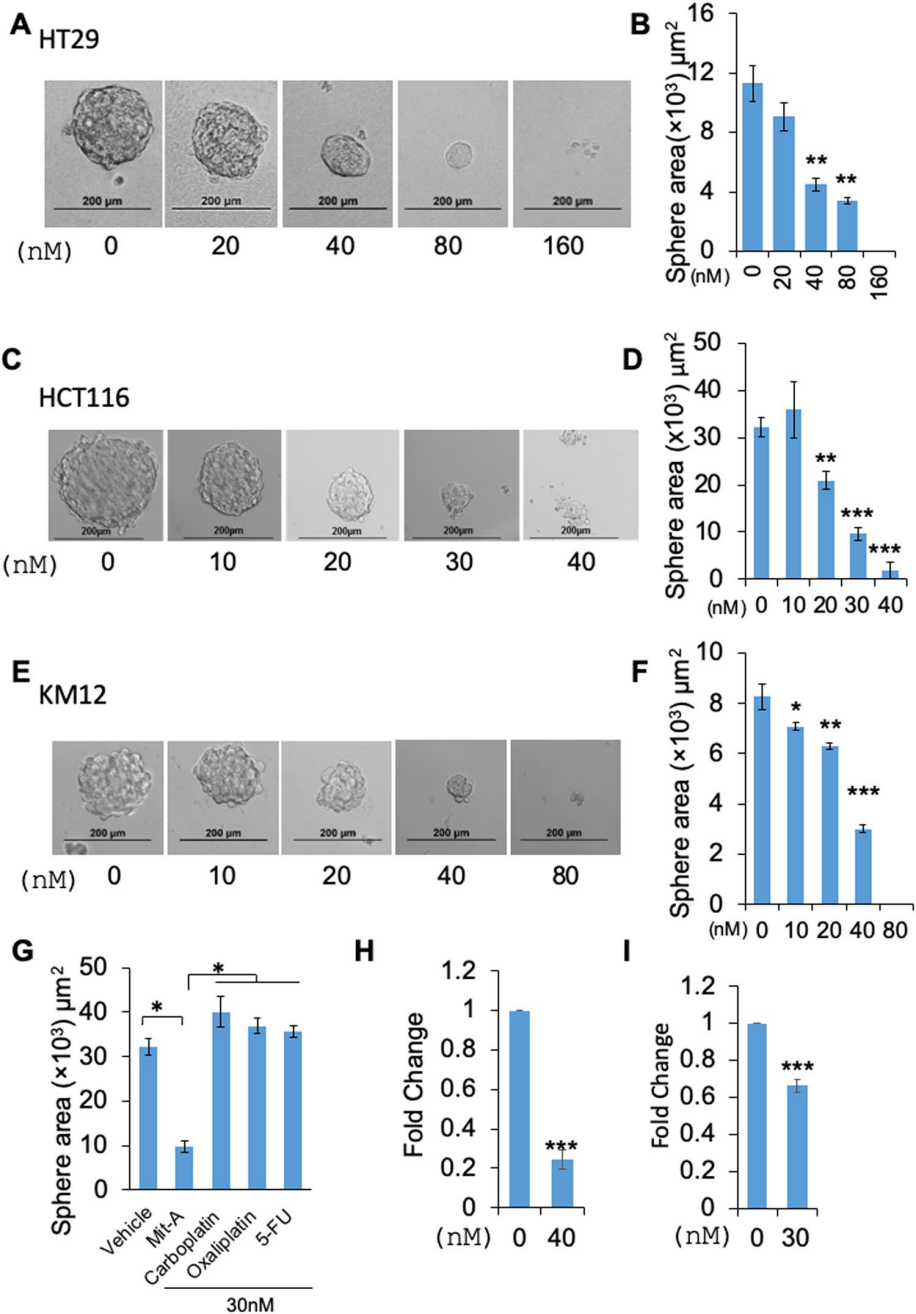
Mit-A - a bona fide anti-CSC target. (**A**,**C**,**E**) HT29, HCT116 and KM12 cells, respectively, were plated in 96 well ultra-low attachment plates and treated with indicated doses of Mit-A for 5 consecutive days to allow the formation of spheres. Images were taken through an inverted microscope and area of spheres was counted (**B**,**D**,**F**, respectively) by using ImageJ software. (**G**) Area of the spheres treated with different anti-cancer drugs, Carboplatin, Oxaliplatin, 5-FU or Mit-A. (**H**,**I**) Total RNA was isolated from HT29 (**H**) and HCT116 (**I**) spheres treated with vehicle and Mit-A with indicated dose, and OCT-4 gene expression analyzed by qPCR. Each data point indicates mean ± SEM. *p < 0.05; **p < 0.01; ***p < 0.001. A representative of two experiments is shown.

To verify whether Mit-A can reduce the stemness properties of CRC cells, we treated HT-29, HCT-116, KM12 and CT26 cells grown in sphere forming media in low attachment conditions for 5 days. In all cell lines, Mit-A significantly reduced the overall size (sphere area) (Fig. 5A–F), number of spheres as well as sphere efficiency (Supplemental Fig. 1) in a dose-dependent manner. In order to compare the efficacy of Mit-A with standard of care chemotherapeutics in suppressing sphere growth, we chose the minimal concentration of Mit-A to significantly reduce the number or size of HCT116 spheres (30 nM) and found that Mit-A was potent in inhibiting sphere growth whereas the standard care drugs Carboplatin, Oxaliplatin and 5-FU could not (Fig. 5G). There was no significant change in the sphere area with these conventional drugs compared to control. Since the spheres are known to highly express the stem cell markers, we also tested whether the reduction of sphere formation ability is due to a decrease in the expression of these markers. qPCR analysis data showed that Mit-A inhibited the colonosphere formation through the inhibition of potential stemness marker OCT4 (Fig. 5H,I) in both HT29 and HCT116 cells, respectively.

### Mit-A can inhibit the *ex vivo* and *in vivo* colon tumor growth

**Figure 6:**
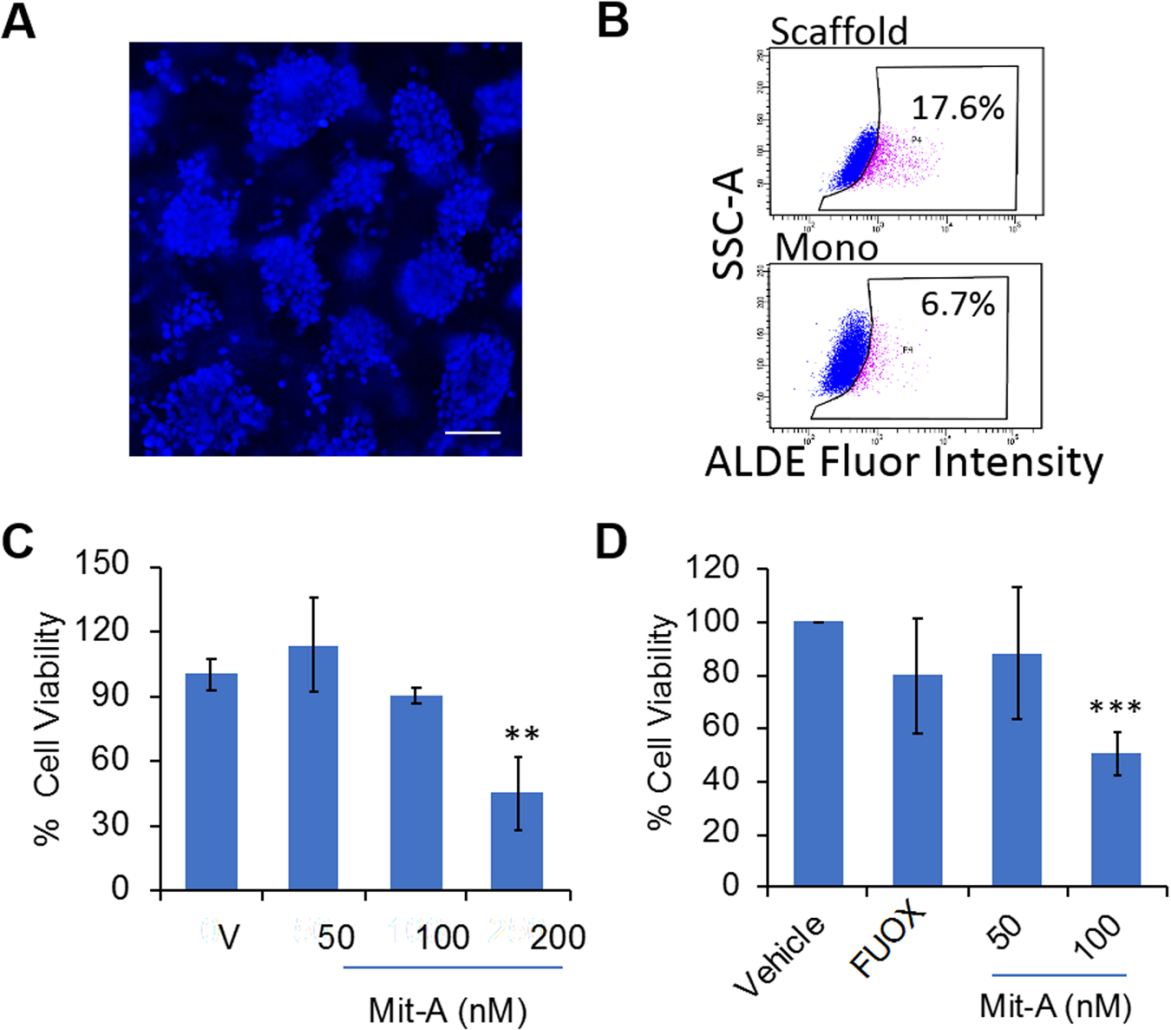
Mit-A inhibits *ex vivo* tumor growth. (**A**) MC38 tumor cells (1 × 10^6^ cells) were injected into the right flank intraperitoneally in C57BL/6 mice. After two weeks, tumors were excised, and single cells isolated from tumor were cultured on scaffolds for 6 days. Cells were stained with Nuc-blue and pictures were taken with a fluorescent microscope, scale bar 100 µm. (**B**) ALDH activity measured using the ALDE Fluor kit (Stem Cell Technologies) in monolayer (bottom panel) compared to scaffold cultured MC38 tumor cells (top panel). (**C**) Day 4 tumoroids were treated with Mit-A for 48 hours. Cell viability was assayed on day 6 by CellTiter-Glo (Promega). (**D**) HT29 tumor cells (3.5 × 10^6^) were injected into the right flank of nude mice in Matrigel. After two weeks the tumors were excised, and single cells isolated from tumor were cultured on scaffolds. Day 4 tumoroids were treated with Mit-A for 48 hours. Cell viability was assayed on day 6 by CellTiter-Glo (Promega). *p < 0.05. A representative of three experiments is shown.

We have previously reported that *ex vivo* culture of tumoroids from tumor biopsy-derived cells retained stromal cells, such as cancer-associated fibroblasts, immune cells and endothelial cells even after 6 days of culture^12,13^. Since tumor microenvironment modulates the responsiveness to drugs *in vivo*, we examined the growth inhibition potential of Mit-A in *ex vivo* MC-38 (murine CRC cells, p53 mutant, K-RAS wild-type, microsatellite instable)^23^ and HT-29 tumoroid growth. To test this, MC38 CRC cells (10^6^ cells/mouse) were implanted subcutaneously in C57BL/6 mice. A single cell suspension of tumors isolated from mice was cultured *ex vivo* on the scaffold, which formed tumoroids (Fig. 6A). Flow cytometry data showed a 2.8-fold increase in ALDH activity in the tumoroids derived from MC38 tumors compared to monolayer (Fig. 6B; 17.6% in tumoroids vs. 6.7% in monolayer). Moreover, Mit-A treatment inhibited the growth of *ex vivo* MC-38 tumoroids in a dose dependent manner (Fig. 6C). Similarly, Mit-A also reduced the growth of *ex vivo* HT-29 tumoroids (Fig. 6D) in a dose-dependent manner.

**Figure 7:**
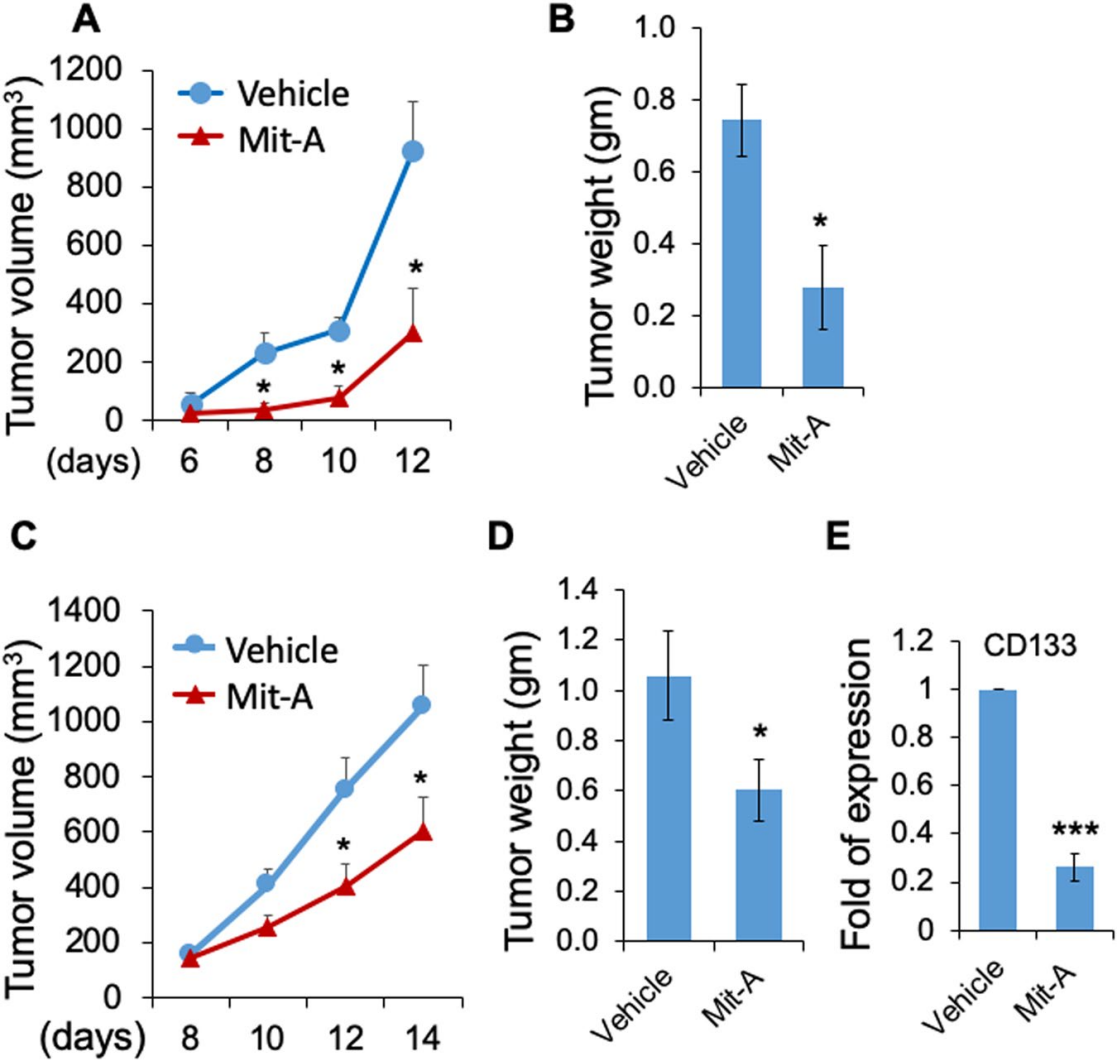
Mit-A inhibits *in vivo* tumor growth. MC38 (1 × 10^6^) (**A**,**B**) and CT26 (5 × 10^5^) (**C**,**D**) tumor cells were injected subcutaneously into the right flanks of C57BL/6 mice (control n = 4, treated n = 5), and BALB/c (control n = 11, treated n = 10), respectively. When tumors are palpable, Mit-A or vehicle was injected intraperitoneally at indicated days after tumor inoculation. Tumor growth was monitored by caliper measurement and mice were sacrificed when tumors reached 10 mm in diameter. Tumor weights were plotted as the mean ± SEM. (**E**) RNA was isolated from vehicle and Mit-A treated tumors and analysed by qPCR (n = 3). *P < 0.05 and ***P < 0.001. A representative of two experiments is shown.

To determine the antineoplastic effect of Mit-A *in vivo*, we implanted mouse tumor cells MC38 and CT26 into C57BL/6 and BALB/c mice, respectively. Intraperitoneal injection of Mit-A of 1 mg/kg/day significantly inhibited the tumor growth and tumor weight in both mice (Fig. 7A–D). The body weight did not change significantly between the groups during the course of the treatment (Supplemental Fig. 2), showing the negligible toxicity of Mit-A on these mice. qPCR analysis also confirmed that Mit-A inhibition of the CT26 tumor growth correlated to the reduced expression of CD133 (Fig. 7E) suggesting that Mit-A targets CSCs *in vivo*.

**Figure 8:**
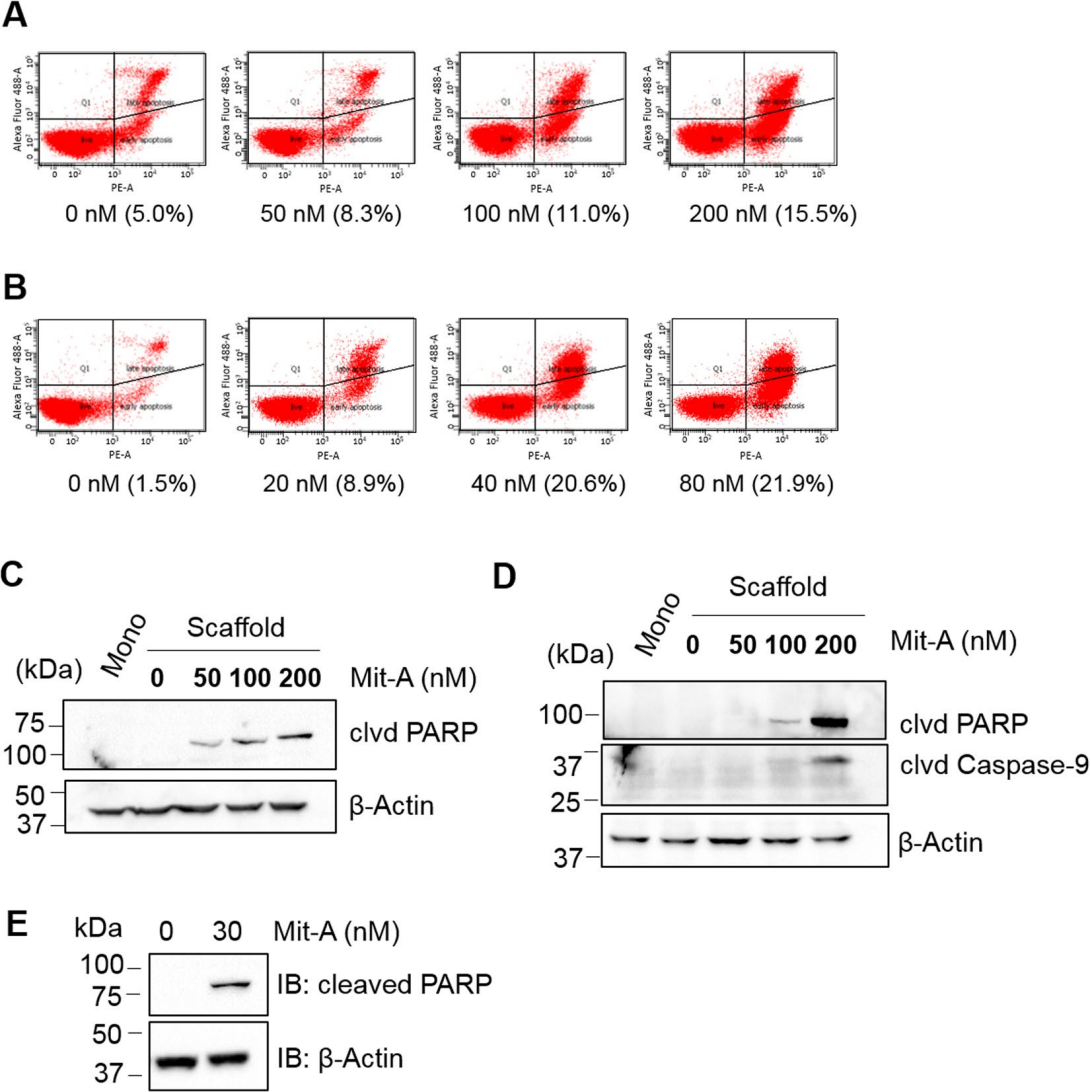
Mit-A induces cell death. (**A**) HT29 and (**B**) HCT116 cells grown on monolayer were treated with Mit-A for 48 hours and flow-cytometry was performed. Experiment was repeated twice and a representative data showing the percentage of cell death (Annexin V+ and Sytox Green+) with different concentration of Mit-A is shown. HT29 (**C**) and HCT116 (**D**) cells were grown on scaffolds for four days and treated with Mit-A for 48 hours at indicated doses. (**E**) HCT116 cells were grown on suspension culture in the presence of Mit-A (30 nM) for 6 days. Proteins were isolated from cells and western blot was performed to detect the expression of indicated proteins in untreated and treated cells. A representative of three experiments is shown.

We next sought to determine the mode of cell death induced by Mit-A. A dose-dependent treatment on HT29 and HCT116 monolayer cells for 48 hours followed by a cell death pathway analysis indicated that Mit-A significantly induces cell death in both cell lines (Fig. 8A,B). To analyze the cell growth suppression mechanism induced by Mit-A, we cultured CRC cells on scaffold and treated with Mit-A for 48 hours. As expected, Mit-A induced PARP1 mediated mitochondrial cell death in CRC cells. In both HT-29 and HCT-116 cell lines, Mit-A induced PARP cleavage in a dose-dependent manner (Fig. 8C,D). Also, in HT-29 cells Mit-A induced cleavage of Caspase-9 suggesting activation of mitochondrial cell death in these cells (Fig. 8D). To analyze whether Mit-A can induce cell death in CSCs, we cultured HCT116 cells in ultra-low attachment plate in suspension culture to induce stem cell growth and treated with Mit-A for 5 consecutive days. We found that Mit-A can kill CSCs by PARP mediated cell death pathway (Fig. 8E). Taken together, these studies suggest that Mit-A can induce apoptosis of both CSC and non-CSC CRC cells.

## Discussion

CRC is often diagnosed at the advanced stage leaving the patient to be treated only by chemotherapy. The conventional treatment of CRC includes 5-FU, FOLFOX, and cisplatin-based treatments, which are not effective when used either alone or in combination^4^. Also, in the advanced stage patients rapidly develop chemoresistance and >50% of patients have a relapse of cancer. Therefore, it is an urgent need to establish new chemotherapeutic drugs that can advance ‘total cancer therapy’, i.e., can effectively kill not only cancer cells, but also drug resistant CSCs that cause tumor relapse. To this end, we have identified Mit-A as a ‘total cancer therapy drug’ and characterized it to show for the first time that it can successfully inhibit CSC proliferation in addition to inhibiting the growth of bulk cancer cells.

Mit-A has been reported to inhibit growth of cancer cells of squamous cell carcinoma^24^, cervical^17^ and prostate cancer^25^ origin, however, its role in inhibiting CRCs has not been investigated heretofore. Additionally, the role of Mit-A in the emerging concept of CSC-targeted treatments has not been studied. A major finding of this study is that we show here for the first time that Mit-A can inhibit both human and mouse CRC growth, irrespective of the driver mutations they carry. Furthermore, Mit-A inhibits CRC growth through the suppression of proliferation of colon CSCs as revealed by reduced expression of CSC markers. Our results demonstrate that Mit-A effectively suppresses CSC growth through stem cell marker inhibition in conventional sphere culture and recently established CRC tumoroid culture on 3-D polymeric scaffold system^12–14^ which rapidly expands CSCs.

CSCs play a pivotal role in CRC progression, metastasis, chemoresistance and cancer relapse. However, there is not a single stem cell marker for CRCs that can be regarded as the most important marker enabling tracking of these cells. Nonetheless, CD133 is considered as an important marker of CRCs since CD133 positive cells have been associated with tumor-initiating properties, whereas CD133 negative cells are not^26^. Additionally, there exist several other CSC markers including LGR5, SOX2, NANOG, OCT4^27,28^. Furthermore, SP1, a transcriptional activator, has been shown to play a crucial role in chemoresistance in CRC^20^. We and others have shown that there is a good correlation between inhibition of CSC marker expression and inhibition of tumor initiation *in vivo* in breast, lung and also CRC^12,29–32^.

Another major finding of our studies is that Mit-A can inhibit the number and growth of both human and mouse CRC cells in sphere cultures that are enriched in CSCs. This sphere growth inhibition was evident from the suppression of the stem cell markers like CD133, LGR5, ALDH. Of note, ALDH1 is one of the major CSC markers^33^. Polymeric 3D scaffold-based cultures provide an excellent tool to expand cancer stemness and amplification of CSCs and such culture could be used to discover new anti-CSC drugs. Cancer stemness can be maintained through repeated passaging of cells isolated from spheres^34^ and similarly the results of our tumoroid cultures showed that cancer stemness increases through successive generations. In addition, we successfully established *ex vivo* tumoroid culture from MC38 and HT29 tumors grown in mice, which allows us to expand the rare CSC population from *in vivo* tumors and simultaneously screen for CSC targets. It is noteworthy that Mit-A was able to effectively suppress the *ex vivo* tumoroid growth of MC38 and HT-29 tumoroids. In addition, we have also shown that recurrent intraperitoneal injection of Mit-A can suppress CRC cell growth in mice. This reduction with marginal insignificant body weight change showed that Mit-A causes minimal toxicity and therefore can be a good therapeutic candidate. These results are consistent with previous reports of Mit-A inhibition of prostate, breast and cervical cancers^17,24,25^.

**Figure 9:**
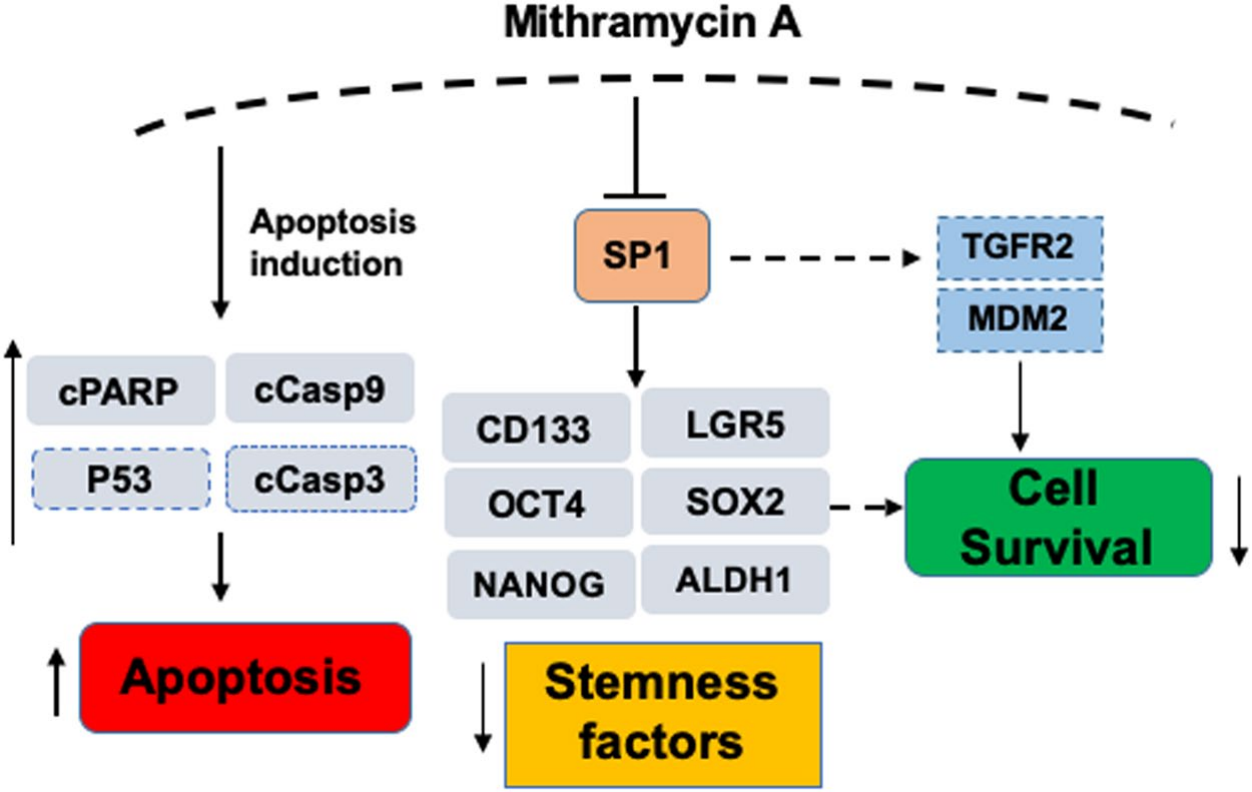
Proposed model of Mit-A action. Proteins shown in this model but not included in our study have been bordered with dashed lines.

In conclusion, we have shown here for the first time that Mit-A induces apoptosis of both CSC and non-CSC CRC cells through inducing the cleavages of PARP and caspase 9 (Fig. 9). Mit-A can inhibit the growth of colorectal CSCs through the inhibition of transcription factors and stem cell markers including but not limited to SP1, CD133, LGR5 and OCT4. An *in silico* analysis by using the Ingenuity Pathway Analysis software by Qiagen Bioinformatics showed that Mit-A can also regulate P53, TGFβ receptor 2 (TGFR2), and MDM2 through SP1, which can have potential impact in cell survival pathway. Although it is still unclear how these proteins could be directly impacted by Mit-A and affect cell survival/death, more studies are warranted to understand the mechanism of Mit-A in suppressing cancer growth. These findings, taken together, suggest that Mit-A could be a potential drug for treating CRC patients.

## Methods

### Antibodies and reagents

Anti-α-actinin (H-2), anti-β-actin (AC-15), and anti-SP1 (E-3, sc-17824) antibodies were from Santa Cruz Biotechnology (Santa Cruz, CA). Anti-cleaved PARP (5625 S), anti-cleaved caspase-9 (9501P) antibodies were from Cell Signaling. Anti-CD133 (ab19898) antibody was from Abcam (Cambridge, MA). Mithramycin A (Sigma, M6891) was purchased from Sigma and was dissolved in DMSO. Accutase (AT-104) was bought from Innovative Cell Technologies. E cadherin (sc-8426) was purchased from Santa CruzBiotechnology, Inc., and α-smooth muscle actin (αSMA) from Abcam.

### Cell culture and drug treatments

HT29, HCT116, SW480 and CT26 were purchased from ATCC. HT29 and HCT116 cells were maintained in McCoy’s 5A medium containing 2 mmol/L L-glutamine, 100 U/mL penicillin, 100 mg/mL streptomycin, and 10% FBS. SW480 and CT26.WT media were cultured in RPMI-1640 medium containing 2 mmol/L L-glutamine, 100 U/mL penicillin, 100 mg/mL streptomycin, and 10% FBS. MC38 cells were kindly provided by Dr. Shari Pilon-Thomas (Moffitt Cancer Center) and were cultured in complete medium as recommended^35^. KM12 cells were obtained from Dr. Bhaumik Patel, Richmond, VA. All cells were maintained in an atmosphere containing 5% CO_2_ and at 37 °C. In addition, cells were routinely checked for mycoplasma contamination. C57BL/6 and BALB/c mice were purchased from The Jackson Laboratory.

### Tumoroid culture

Polymeric nanofiber scaffold was prepared as previously described^12^. Scaffolds were sterilized in ethanol and prepared for tumoroid culture as described^12^. Cell lines were obtained from ATCC. Cells were cultured in a humidified incubator at 37 °C in a 5% CO_2_ atmosphere (Thermo Fisher). 3D Tumoroid formation was assessed using fluorescent microscopy (Olympus BX51) after nuclear staining with Nuc Blue dye (Thermo Scientific). For immunofluorescence (IF) staining the tumoroids were washed with PBS and incubated with E-cadherin (sc-8426) (1:500) and α-smooth muscle actin (SMA) primary antibodies (1:500) overnight separately and then with secondary antibodies (donkey anti-mouse (594) 1:1000 and donkey anti-goat (488) respectively). The monolayers were stained the next day of plating and were stained similarly. The images were taken in confocal microscope (Olympus FV1200 Laser Scanning Confocal Microscope).

### Scanning electron microscope (SEM)

SEM was performed using methods as described previously^12^. Briefly, the day 4 tumoroids on scaffolds were fixed in 4% formaldehyde and then sodium cacodylate and osmium tetroxide were added to the scaffold with intermediate shaking at each step for 5 min at RT. Following the above procedure, 30% and then 70% ethanol was added after which the scaffolds were incubated overnight. Next day the scaffolds were submerged in HMDS and 50% ethanol and dried to air following which they were mounted for sputter coating.

### Sphere assay

To measure the sphere formation ability, cells were trypsinized and were plated at a density of either 100 cells or 200 cells per well in a 96 well ultra-low attachment surface plate (Corning Inc. 3474) in serum free DMEM/F-12K and DMEM High Glucose (1:1) media containing 0.004 gm/ml BSA, epidermal growth factor (100 ng/ml), fibroblast growth factor (20 ng/ml), insulin (5 µg/ml) and B27. Cells were incubated in 5% CO_2_ and at 37 °C and treated with Mithramycin A for 5 days. Sphere pictures were taken manually using a bright field microscope and total area of the spheres was calculated using Image J software. Each experiment represents a combination of three replicates (n = 3). The data was repeated at least twice.

### Cell viability assay

Cell growth was quantified using CellTiter-Glo® Luminescent Cell Viability (Promega, G7572) assay. For monolayer culture, cells were plated in a 96 well plate and treated the next day with Mithramycin A as indicated (n = 4). For scaffold culture, cells were plated in a 96 well plate in 50 µl volume to stabilize cells on the scaffold (n = 4). Next day, 150 µl fresh media was added. On day 4 of plating, cells were treated with Mithramycin A. In both cases, 48 hours after treatment, cell titer glow reagent was added with media (1:1 ratio) according to the manufacturer’s protocol and the luminescence signal was read by an illuminometer in an opaque plate.

### Western blot analysis

Whole cell lysates were extracted using lysis buffer and equal amounts of proteins were loaded in an 8–10% SDS-PAGE gel. Samples were transferred from the gel onto a nitrocellulose membrane (Bio-Rad, 162-0112) and incubated with 5% skim milk for an hour to block the membrane. Indicated antibodies were used for overnight incubation at 4 °C and the blots were developed using ECL substrates after HRP-conjugated secondary antibody incubation. β-Actin or α-Actinin blots were run for internal control.

### Flow cytometry

To measure the cell marker expression associated with stemness, flow cytometry was performed. Cells from both monolayer and scaffolds were grown and treated with either vehicle or Mithramycin A as indicated. A single cell suspension was obtained by treating the cells with Accutase. Cell marker expression was observed by using Aldefluor kit (Stem Cell Technologies, 01700), anti-CD133 antibody (Miltenyil 130-113-188) and anti- leucine-rich repeat-containing G-protein coupled receptor (LGR5) (Abcam, 75850) antibody. Expression of the markers was analyzed with a BD FACS Canto II fluorescent activated cell sorter.

### qRT-PCR

To quantify the gene expression, cells were either untreated or treated with Mithramycin A with indicated concentration for 48 hours. Total RNA was isolated using Trizol (Life Technologies). Residual DNA (if present) were removed by treating 1 µg RNA by dNase I (Invitrogen, 18068). cDNA was prepared from 1 µg of RNA using Maxima Enzyme and 5X. The reaction Mix (Thermoscientific). Quantitative real-time PCR reactions were performed using cDNA in CFX384 Touch™ Real-Time PCR Detection System (Bio-Rad). Reaction mixture was prepared in a total 5 µl volume containing 2.5 µl 2X all-in-one qPCR master mix (Genecopoeia, QP001), 0.5 µl of forward and reverse primers (see Table 1, Supplementary data) and 1 µl of cDNA. Reactions were run in triplicates (n = 3) in following cycles: 95 °C for 3 min, followed by 45 cycles of 95 °C for 10 s, 60 °C for 1 min and 72 °C for 15 s. β-Actin was run as an internal control for human and mouse genes, respectively. ΔΔ^−Ct^ values were calculated to measure each gene expression change.

### Cell death analysis

Cells were seeded in a 6-well plate (1.5 × 10^5^ and 1 × 10^5^ cells per well, respectively) and incubated overnight. Cells were incubated with increasing concentration of Mithramycin A for 48 h. Cell death analysis was performed by an Annexin-V- PE Apoptosis detection kit (eBioscience 88-8102-74). Cells were stained with AnnexinV-PE and SYTOX GREEN concurrently for 15 min at RT in dark 400 µL of 1X binding buffer was added prior to analyze the cells in the flow cytometer. AnnexinV and SYTOX GREEN double positive cells were considered as late apoptotic or necrotic cells.

### *In vivo* experiments

6–8 weeks old C57BL/6 and BALB/c mice were injected subcutaneously in the right flank with MC38 (1 × 10^6^) and CT26.WT cells (5 × 10^5^), respectively. Intraperitoneal injections (1 mg/kg) of Mithramycin A were started seven days after tumor injection and were given daily. Tumor volume was calculated using the formula (0.5 × Length × Width^2^) where width was the shorter dimension. Mice body and tumor weight were measured every 48 hours after Mithramycin A injection started. On day 14, mice were euthanized, and tumors were isolated for further studies. All animal work was approved by and performed in accordance with the policies of the University of South Florida Institutional Animal Care and Use Committee.

### Statistical analysis

All quantitative data were analyzed through mean ± S.E.M unless otherwise stated. A p value < 0.05 was considered statistically significant.

## Supplementary information

Supplementary Info
