# Supplementary material for: Mithramycin A Inhibits Colorectal Cancer Growth by Targeting Cancer Stem Cells
Source: Sci Rep. 2019 Oct 23;9:15202. doi: 10.1038/s41598-019-50917-3 (PMC6811578; doi:10.1038/s41598-019-50917-3)

Supplementary Information

Mithramycin A Inhibits Colorectal Cancer Growth by Targeting Cancer Stem Cells

Waise Quarni, Rinku Dutta, Ryan Green, Sandhyabanu Katiri, Bhaumik Patel, Shyam S Mohapatra and Subhra Mohapatra

Table 1: Primers used for qRT-PCR in this manuscript.

Mouse Primers	Forward	Reverse
Beta Actin	GGGGTGTTGAAGGTCTCAAA	AAATCTGGCACCACACCTTC
SOX2	GAAGCGCCTAACGTACCACT	TTAACGCAAAAACCGTGATG
CD133	TTGTTCTGGTTCGGCATAGG	CTGAGTCTCCACCAGGTTTC
LGR5	TGCCCATCACACTGTCACTGT	CACCCTGAGCAGCATCCTG

Human Primers	Forward	Reverse
Beta Actin	GGGGTGTTGAAGGTCTCAAA	TTCTACAATGAGCTGCGTGTG
OCT4	TCCAGGTTTTCTTTCCCTAGC	TGRACTCCTCGGTCCCTTTC
SOX2	GCAAGAAGCCTCTCCTTGAA	GCTAGTCTCCAAGCGACGAA
NANOG	CTCGCTGATTAGGCTCCAAC	CAGTCTGGACACTGGCTGAA
CD133	CAAATGTGGTGGAGAAATGC	GTGATTTGCCACAAAACCAT
LGR5	TCAGTCAGCTGCTCCCGAAT	CGTTTCCCGCAAGACGTAAC
ALDH1	CACGGGCCTCCTCCACATT	AGGGGCAGCCATTTCTTCTCA
SNAIL	AGACGAGGACAGTGGGAAAG	AGATCCTTGGCCTCAGAGAG

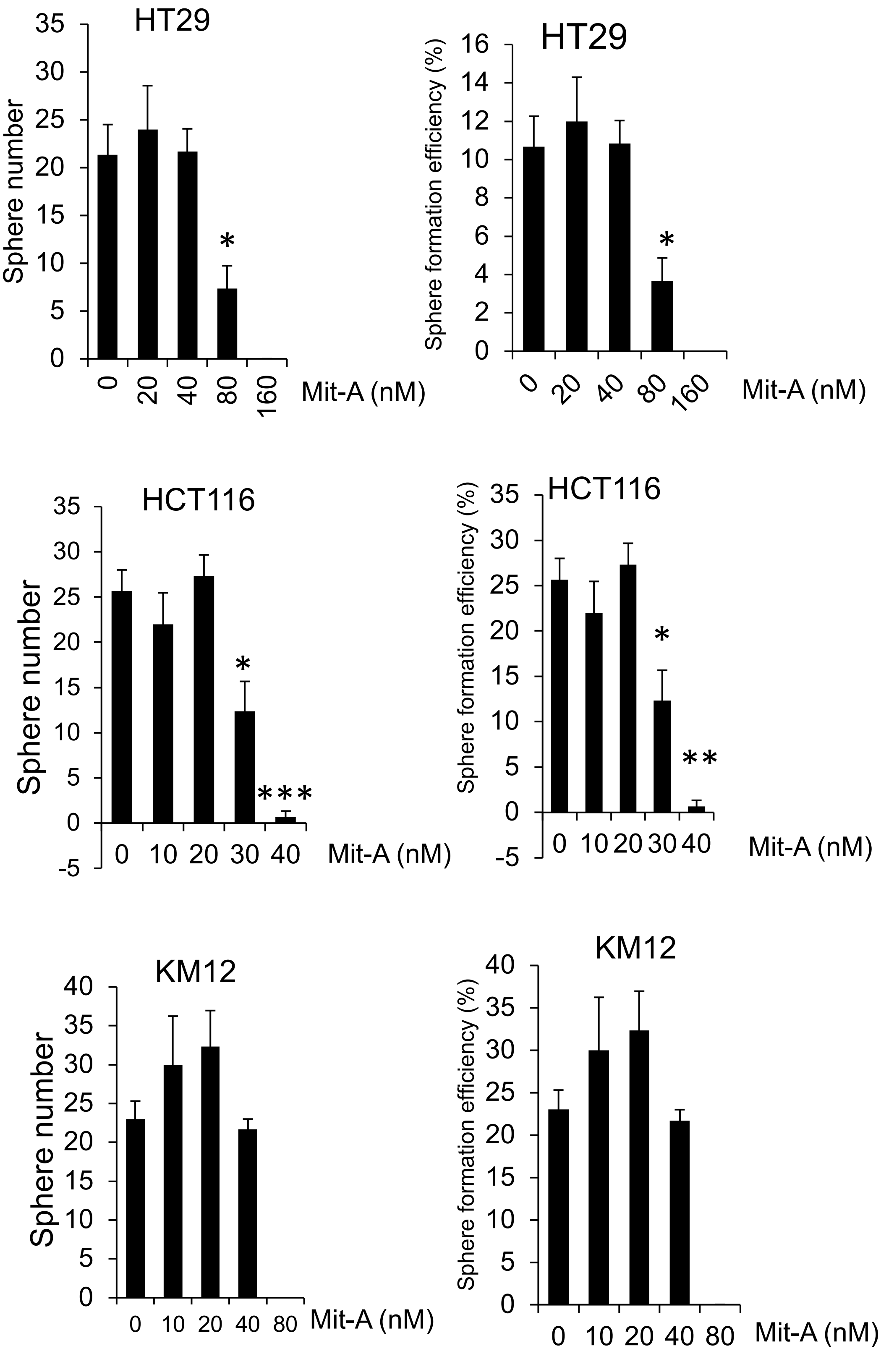

<u>Supplementary figure 1</u>: HT29, HCT116 and KM12 cells were plated in 96-well ultra-low attachment plates (200, 100 and 100 cells per well, respectively) in stem cell culture media. Cells were treated with Mithramycin A with indicated doses for 5 consecutive days and cell numbers were counted with microscope. Sphere formation efficiency was measured as the number of spheres formed per 100 cells plated in each well.

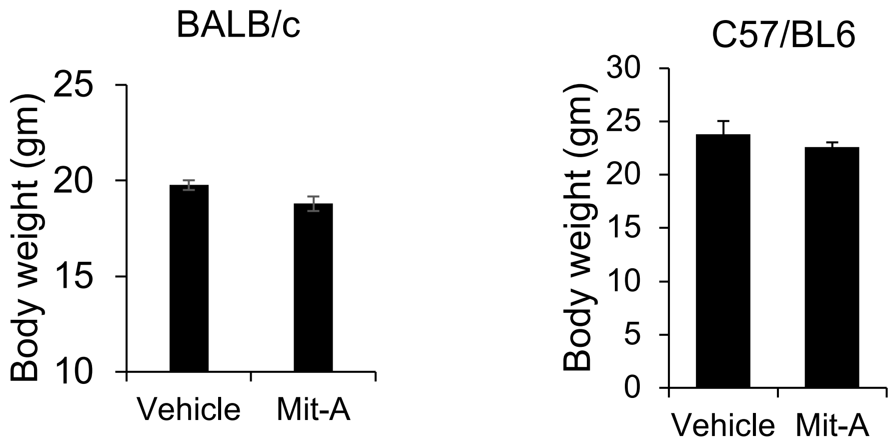

<u>Supplementary figure 2</u>: Body weight of the BALB/c and C57/BL6 mice treated with either Vehicle (DMSO) or Mithramycin A (Mit-A), as described in figure 6. Body weight was measured starting the day of tumor cell injection into the flank and measured every other day until they were sacrificed.

Fig. 1G (uncropped blots)

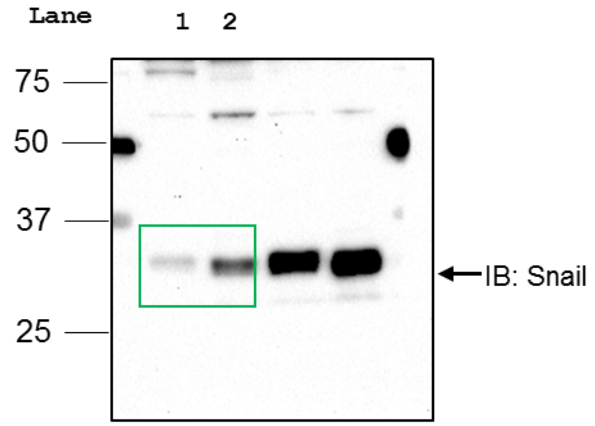

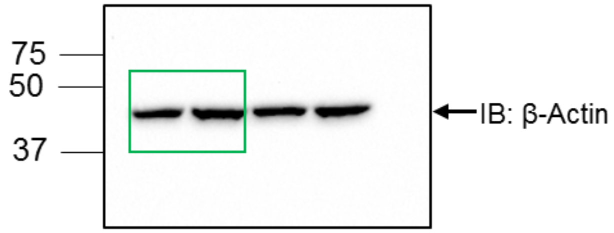

Fig. 1H (uncropped blots)

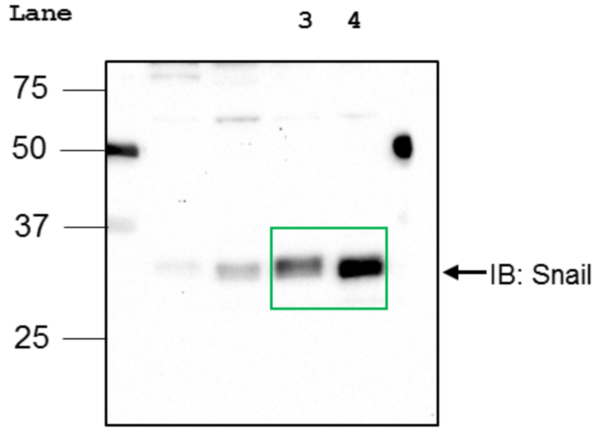

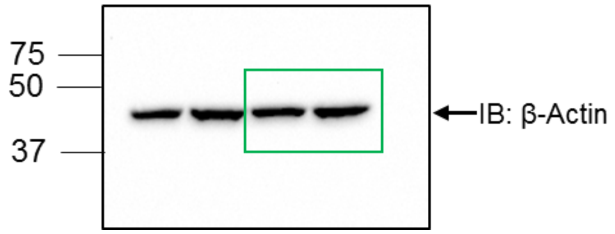

Fig. 5D (uncropped blots)

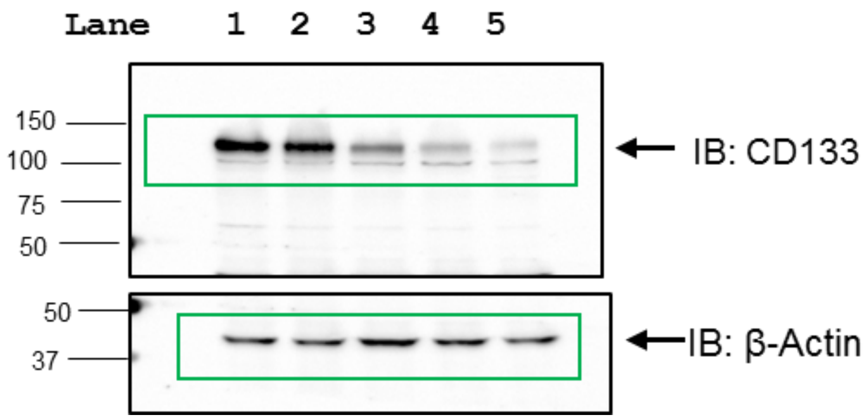

Fig. 5E (uncropped blots)

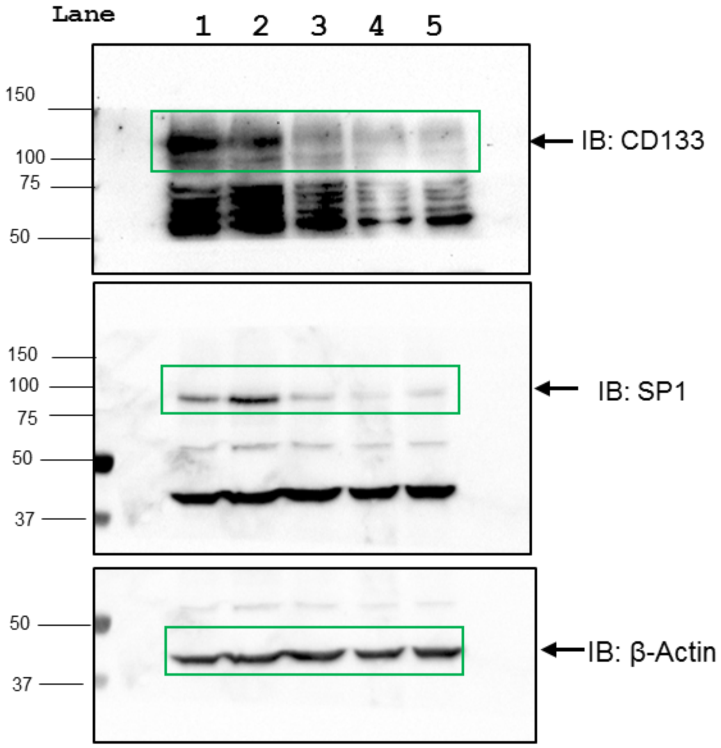

Fig. 5F (uncropped blots)

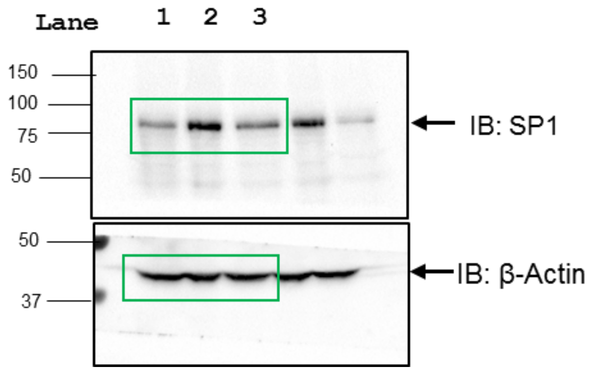

Fig. 8C (Uncropped Blot)

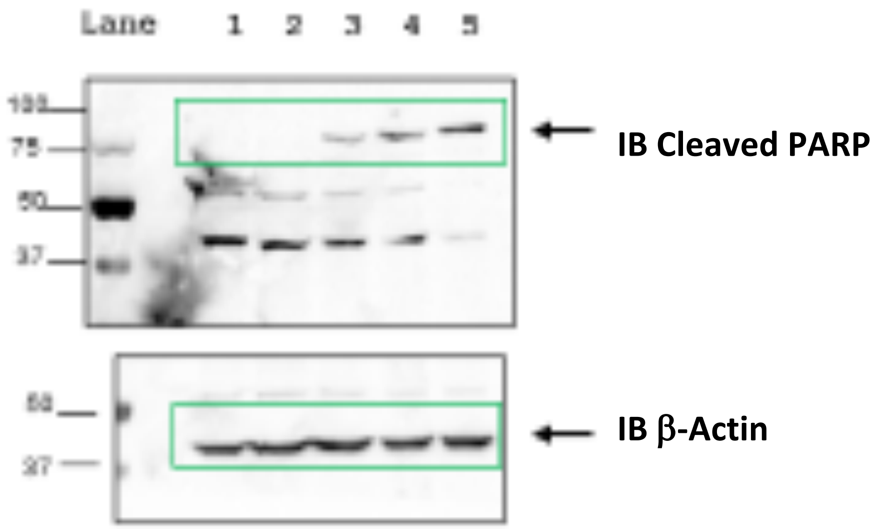

Fig. 8D (uncropped blots)

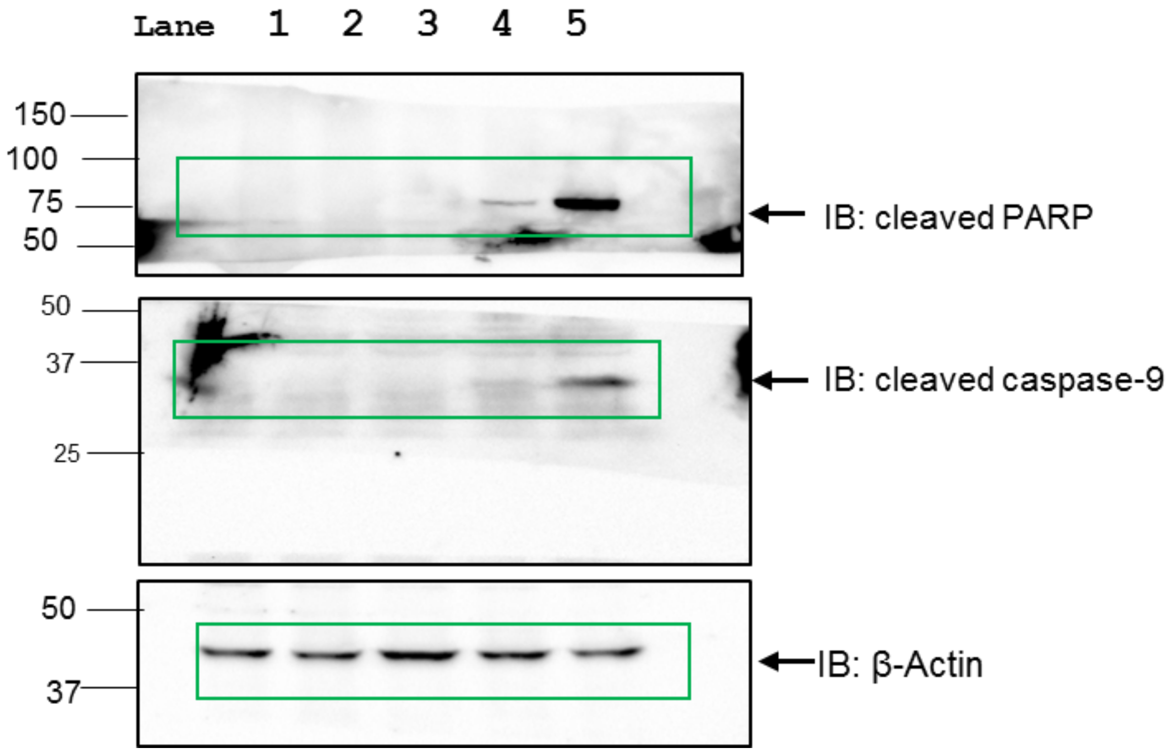

Figure 8 E (uncropped blot)

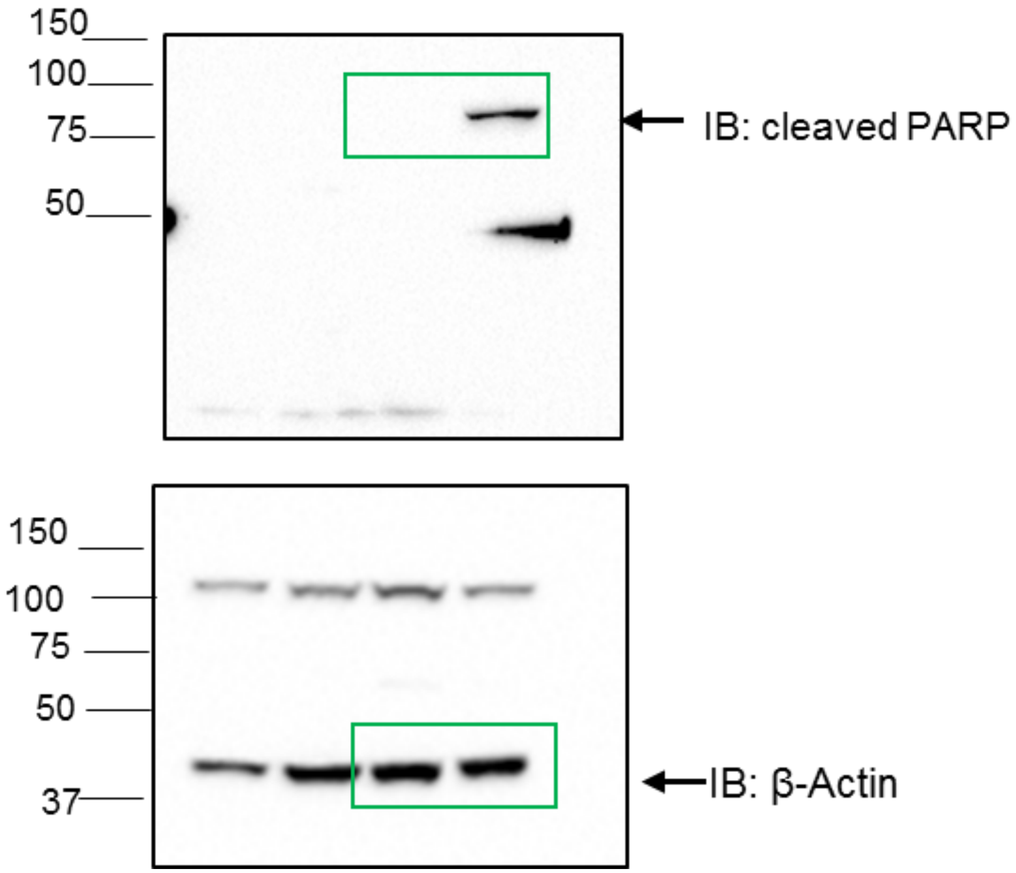

Supplement: Supplementary file 1 — Supplementary Info [file 41598_2019_50917_MOESM1_ESM.pdf]
